# The Relationship Between Metabolic Syndrome and Left Ventricular Mass Index in Obese Children

**DOI:** 10.4274/jcrpe.v3i3.26

**Published:** 2011-09-09

**Authors:** Mehmet Emre Atabek, Beray Selver Eklioğlu, Esra Akyüz, Derya Çimen

**Affiliations:** 1 Selçuk University School of Medicine, Department of Pediatric Endocrinology and Diabetes, Konya, Turkey; 2 Selçuk University School of Medicine, Department of Pediatrics, Konya, Turkey; 3 Selçuk University School of Medicine, Department of Pediatric Cardiology, Konya, Turkey; +90 332 223 63 50 berayselver@hotmail.com growth

**Keywords:** obesity, metabolic syndrome, cardiovascular disease, left ventricular mass index, children

## Abstract

**Objective:** To investigate the relationships between metabolic syndrome (MS), other metabolic features and left ventricular mass index (LVMI) in a population of obese children and adolescents with MS.

**Methods:** Two hundred and eight obese children and adolescents (119 females and 89 males, mean age: 11.9±2.7 years) and control subjects (24 females and 26 males, mean  age: 11.4±2.9 years) were enrolled in the study. The insulin sensitivity index and LVMI  were  determined. The International Diabetes Federation criteria were used to diagnose MS.

**Results:** The obese patients were divided into MS group (n=55) and non-MS (n=153) group. The values of LVMI in the MS group were significantly higher than those in the non-MS group (p=0.014). The present LVMI cut-off point of 33g/m2 for the diagnosis of MS yielded a sensitivity of 97% and a specificity of 98%. LVMI was found to be positively correlated in univariate analysis with height, weight, body mass index (BMI) SDS, fasting insulin level, homeostasis model assessment of insulin resistance (HOMA-IR) and fasting glucose to insulin ratio (FGIR) and negatively correlated with quantitative insulin sensitivity check index (QUICK-I).

**Conclusions:** We suggest that our optimal LVMI cut-off value for identifying MS may be considered as a sensitive index in screening obese children and adolescents for pediatric MS. Assessment of LVMI in obese children and adolescents may be used as a tool in predicting the presence of MS and its associated cardiovascular risks.

**Conflict of interest:**None declared.

## INTRODUCTION

The prevalence of obesity has increased dramatically in children and adolescents, in both  developed and developing worlds, becoming an important medical problem. Many of the outcomes of obesity have traditionally been viewed as problems of adulthood. However, it has become clear that many of these abnormalities may start in childhood and adolescence (1,2,3,4).

  Obesity affects cardiovascular parameters such as left ventricular (LV) mass and cardiac function as well as metabolic parameters such as insulin levels and glucose tolerance (5). These latter variables are directly associated with hypertension, although the mechanism is not fully understood. Epidemiologic evidence has shown that insulin resistance is an independent risk factor for atherosclerosis and coronary heart disease and is also a major cause of type 2 diabetes mellitus (T2DM) (6,7). Thus,  the insulin-resistance syndrome may be considered the hallmark for the development of both diabetes and cardiovascular disease (8). The metabolic syndrome (MS) is a cluster of atherogenic risk factors including abdominal obesity, hypertension, insulin resistance,  dyslipidemia, and a proinflammatory as well as a prothrombotic state (9,10). It has been previously reported that MS is related to abnormal LV geometry and function in nondiabetic adults with a high prevalence of obesity, and that increased blood pressure (BP) is the MS component most strongly associated with markers of pre-clinical cardiovascular disease even in the absence of traditionally defined hypertension (11,12). 

MS and T2DM prevalences among obese adolescents are quite high in the urban area of Konya, a city in the central Anatolian region of Turkey (1,13). In a previous study, we found that the prevalence of MS was 27.2% among obese children and adolescents with a significantly higher rate among the adolescents aged 12–18 years (37.6%) than among obese children aged 7–11 years (20%) (1). To date, limited information is available on whether the presence of MS is associated with significant cardiac abnormalities in obese children and adolescents, or whether the impact of MS on cardiac phenotype is independent of the single  components of the syndrome.  To our knowledge, there have been no comprehensive studies regarding the relationship between MS and LV mass index (LVMI) during childhood. The aim of our study was to investigate the relation between MS and LVMI in a population of obese children and   dolescents with MS as well as the relationships between other metabolic features with LVMI. 

## MATERIALS AND METHODS

208 obese children and adolescents (119 females and 89 males) were recruited from the group of  obese children  attending the outpatient clinic of the Pediatric Endocrinology Unit of Selcuk University Hospital in Konya, Turkey between December 2006 and December 2008. Obese children were included in the study if they were 7-17 years of age and had BMI≥95th percentile for age and gender based on the standards of the Centers for Disease Control and Prevention (14).  

The mean age of the patients was 11.9±2.7 years (range:  7-17 years) and the mean body mass index (BMI) was 29.1±4.8 kg/m2.  The control subjects were recruited from a population of nonobese healthy children and adolescents who presented to the hospital for minor illnesses. The control group consisted of 24 females and 26 males aged 6-17 years. Their mean age was 11.4±2.9 years and their mean BMI was 17.4±2.7 kg/m2. Blood samples from the control group were collected at the time of their clinical evaluation. Children were excluded if they had a major illness including T1DM or T2DM, took medications, or had a condition known to influence body composition, insulin action, or insulin secretion (e.g. glucocorticoid therapy,  hypothyroidism, Cushing’s disease). All subjects were in good health and had normal thyroid functions. The study was approved by the local ethics committee of Selcuk University. Signed informed consent was obtained from each subject over 12 years of age, and informed parental consent was also obtained for all children regardless of age. Each child underwent a complete physical examination, including anthropometric measurements. Height was measured to the nearest 0.5 cm on a standard height board and weight was determined to the nearest 0.1 kg on a standard physician’s beam scale with the subject dressed only in light underwear and no shoes. BMI was calculated as weight (in kilograms) divided by height (in meters) squared. BP was measured with a standard mercury  sphygmomanometer after the subjects had rested at least 10 minutes. Waist circumference was measured at the level of the umbilicus with the patient standing and breathing normally. Hip circumference was measured at the level of the iliac crest. Waist/hip and waist/height ratios were calculated from  the waist and hip circumference measurements. Waist  measurements were evaluated using the percentile curves for waist circumference of healthy Turkish children aged 7-17 years reported by Hatipoglu et al (15).   

**Blood Samples and Oral Glucose Tolerance Test**

After a 3-day high-carbohydrate diet (300 g/d) and an overnight fast, a standard oral glucose tolerance test (OGTT) (1.92 g/kg or a maximum of 82.5 g of glucose) was performed in all subjects in the study group.  The initial venous blood  samples were taken in the morning between 07.30 and 09.30, after the children had fasted overnight. Following this initial sampling (0 minute), venous blood samples were obtained at 30, 60, 90, and 120 minutes  to measure plasma glucose  and insulin levels.  Venous blood samples were also obtained in the control group following an overnight fast, but the control group was not subjected to OGTT, for ethical reasons.  After clotting, the serum was separated and immediately analyzed. Glucose was determined by the glucose oxidase method. Plasma concentrations of total cholesterol, high- density lipoprotein (HDL) cholesterol, low-density lipoprotein (LDL) cholesterol, triglycerides, apolipoprotein A (Apo-A) and apolipoprotein B (Apo-B) were measured using  routine  enzymatic methods with Olympus 2700 Analyzer (Olympus Diagnostica GmbH, Hamburg, Germany). Insulin levels were measured by a chemiluminescence immunoassay (Immulite, Diagnostic Products, Los Angeles, CA).  

**Measurement of Insulin Sensitivity Check Indices**

Insulin indices were derived from fasting blood samples and samples obtained during the OGTT. The homeostasis model assessment of insulin resistance (HOMA-IR), quantitative insulin-sensitivity check index (QUICK-I), and fasting glucose to insulin ratio (FGIR) were derived as estimates of insulin  resistance. FGIR was calculated as fasting glucose  concentration (mg/dL) /fasting insulin concentration (μU/mL). HOMA-IR was calculated as fasting insulin concentration (μU/mL) x fasting glucose concentration (mmol/L)/22.5 (15). QUICK-I was calculated as 1/ [(log fasting insulin concentration (μU/mL) + log fasting glucose concentration (mg/dL)] (16,17). The total plasma glucose response and insulin secretion were evaluated from the area under the response curve (AUC)  estimated by the trapezoid rule.  

**Echocardiographic Measurements  **

A Philips Sonos 5500 system with a 5 MHz transducer ultrasonic imager was used for echocardiographic  assessments. Participants were examined in the left lateral decubitus position and images were acquired at passive end-expiration to minimize global cardiac movement from  standard parasternal long axis and apical planes, by the same expert operator. The M-mode echocardiographic study of the left ventricle was performed under 2D control. The ventricular septal and posterior wall thicknesses at end-diastole, and LV end-diastolic and end-systolic dimensions were determined from M-mode echocardiogram according to the American Society of Echocardiography recommendations (18). The LVM was calculated using the formula of Devereux et al (19) by the following equation: LVM = 0.80 [1.04 x (interventricular septal thickness + posterior wall thickness + end-diastolic diameter) 3 - (end-diastolic diameter)3] + 0.6. The LVMI was calculated as LVM divided by the body surface area. 

**Definitions**

Based on the MS criteria proposed by the International Diabetes Federation (IDF), patients were diagnosed as having MS  when their waist circumference was ≥90th and when at least two of the following factors were present (20): (1) raised concentration of triglycerides: ≥150 mg/dL (1.7 mmol/L) or receiving specific treatment for high triglycerides; (2) reduced concentration of HDL cholesterol: <40 mg/dL (1.03 mmol/L) or receiving specific treatment for this lipid abnormality; (3) raised BP:  Systolic BP ≥130 mmHg or diastolic BP ≥85 mmHg or receiving treatment for previously diagnosed hypertension; and (4) raised fasting plasma glucose concentration 100 mg/dL (5.6 mmol/L) or known T2DM. The obese patients were divided into MS group (n=55) and non-MS (n=153) group according to the IDF consensus definition of MS in children and adolescents (20). 

**Statical Methods**

The data were expressed as means±SD. The Kolmogorov-Smirnov test was applied separately for males and females to check the normality of the variables. Differences in the means of variables were evaluated using both parametric and nonparametric tests depending on the distribution of the variables. Any variables that were not normally distributed were log transformed before data analysis. Statistical correlation was assessed using the Pearson’s test (r). Separate relationships between LVMI and insulin sensitivity indices (HOMA-IR, FGIR and QUICK-I) were also examined after adjustment for age, sex, BMI-SDS, systolic and diastolic BP, total cholesterol, LDL cholesterol, HDL cholesterol, triglycerides, Apo-A and Apo-B,  using general linear regression models (backward analysis). Statistical significance was taken as p<0.05. All statistical analyses were performed using the Statistical Package for Social Sciences (SPSS/Windows version 13.0, SPSS Inc., Chicago, IL). We performed a receiver operating characteristic (ROC) curve analysis in the SPSS version 11.0 to obtain a ROC plot and a complete sensitivity/specificity report. A ROC curve is a graph that plots the true positive rate in function of the false positive rate at different cut-off points. The results of  ROC curve analysis displayed the average value of sensitivity for all possible values of specificity and the average value of specificity for all possible values of sensitivity. The cut-off points, sensitivity and specificity calculations for indices have been based on LVMI with ROC curve analysis.

## RESULTS

The characteristics of the study population are shown in Table 1. Both the obesity group and control group showed no significant difference in terms of age. Subjects in the obese group had a significantly higher BMI-SDS, systolic and diastolic BP, waist/hip and waist/height ratios. As shown in Tables 2, total cholesterol, LDL cholesterol, Apo-B and triglyceride levels were significantly elevated in obese children, whereas the  concentration of HDL cholesterol was only slightly lower than that in the controls. The values of LVMI were significantly  higher in the obese group than in controls (p=0.033). Obese subjects had significantly elevated HOMA-IR compared to the control group (p<0.001), whereas FGIR and QUICK-I  measurements in obese children were significantly lower than in the control group (p<0.001).

 The MS group had a significantly higher BMI, BMI-SDS, systolic and diastolic BP, waist circumference (Tables 3),  triglyceride levels, HOMA-IR, FGIR and fasting insulin levels than the non-MS group (p<0.001), whereas HDL cholesterol and Apo-A levels of children with MS were lower than those of  non-MS group. The non-MS group had a significantly lower QUICK-I than the control group (p<0.001). The values of LVMI in the MS group were significantly higher than those in the non-MS group (p=0.014) (Table 4).

  Table 5 shows the correlations between LVMI and other measurements in obese children. When LVMI was considered as a continuous variable in the whole population of obese children, it was found to be positively correlated in  univariate analysis with BMI-SDS (r=0.262, p<0.001), waist  circumference (r=0.264, p<0.001), systolic BP (r=0.443, p<0.001) and diastolic BP (r=0.436, p<0.001), triglycerides (r=0.399, p<0.001), fasting insulin levels (r=0.367, p<0.001), HOMA-IR (r=0.341, p<0.001) and FGIR (r=0.351, p<0.001) and negatively correlated with HDL cholesterol (r=-0.261, p<0.001), Apo-A (r=-0.223, p=0.015), QUICK-I (r=–0.278, p<0.001). There was no significant relationship between LVMI and clinical and laboratory parameters in controls (data not shown).  

Table 5 also depicts the correlations between LVMI and other measurements in the MS group. When LVMI was considered as a continuous variable in the whole population of the MS group, it was found to be positively correlated in univariate analysis with height (r=0.278, p=0.023), weight (r=0.62, p=0.004), BMI-SDS (r=0.360, p=0.005), fasting insulin level (r=0.325, p=0.009), HOMA-IR (r=0.319, p=0.010) and FGIR (r=0.311, p=0.013) and negatively correlated with QUICK-I (r=–0.331, p=0.008). 

 In the regression analysis, BMI (b=0.397, p=0.002) and HOMA-IR (b=0.373, p=0.004) were positively correlated with LVMI even after adjusting for age, sex, BMI-SDS, systolic BP/diastolic BP, total cholesterol, triglycerides, LDL cholesterol and HDL cholesterol as co-factors with the total variance explained being 25.7% (Table 6), but QUICK-I and FGIR  were not significantly correlated with LVMI.  When LVMI was considered as a continuous variable in the whole population of the non-MS group, it was found to be positively correlated in univariate analysis with height (r=0.278, p=0.023), weight (r=0.62, p=0.004), BMI-SDS (r=0.360, p=0.005) and systolic BP (r=0.213, p=0.006) and negatively correlated with QUICK-I  (r=-0.331, p=0.008). In the regression analysis, BMI (b=0.498, p<0.001) was positively correlated with LVMI even after  adjusting for age, sex, BMI-SDS, systolic BP/diastolic BP, total cholesterol, triglycerides, LDL cholesterol and HDL cholesterol as co-factors with the total variance explained being 15.7%, but HOMA-IR, QUICK-I and FGIR were not significantly correlated with LVMI (data not shown).  

**Figure 1:**
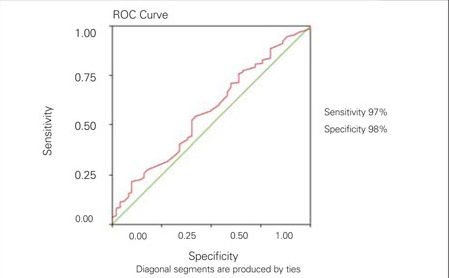
Figure 1. Cut-off points, sensitivity and specificity of LVMI according to metabolic syndrome ROC: receiver operating characteristics, LVMI: left ventricular mass index

The cut-off point for MS, along with sensitivity and  specificity values, is demonstrated in Figure 1. The present LVMI cut-off point of 33 g/m2 for the diagnosis of MS yielded a sensitivity of 97% and a specificity of 98%.

**Table 1:**
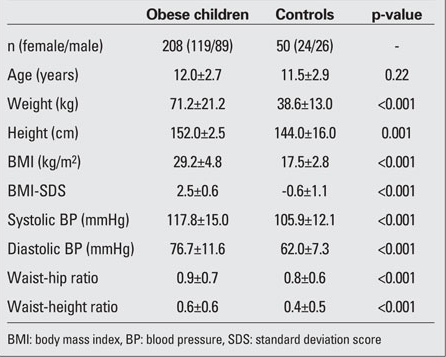
Table 1. Clinical characteristics of the study population  (mean±SD)

**Tables 2:**
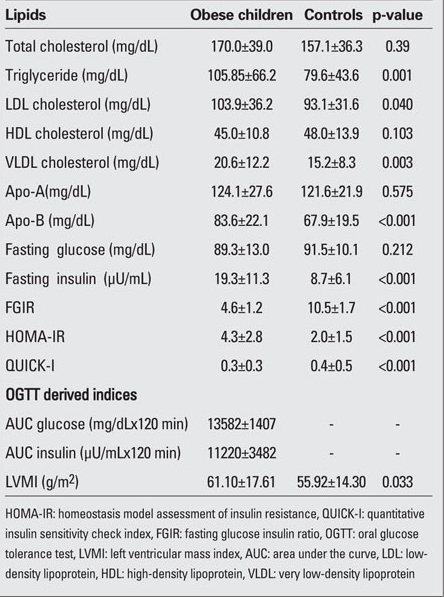
Table 2. Laboratory findings of the study population (mean±SD)

**Tables 3:**
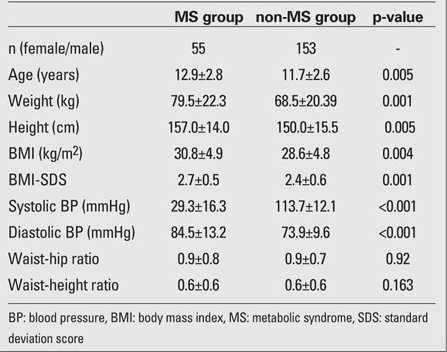
Table 3. Demographic and clinical data of the two subsets with and without MS

**Table 4:**
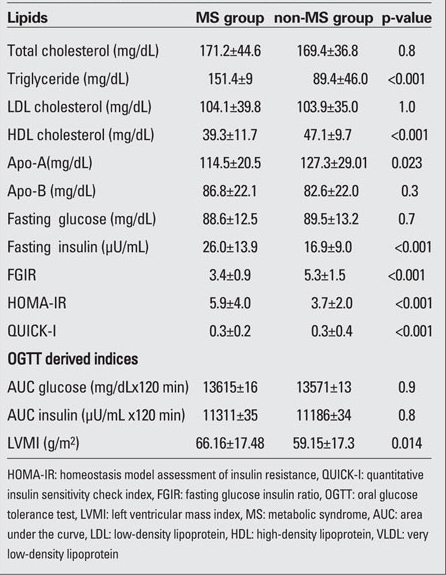
Table 4. Laboratory findings of the two subsets with and without MS

**Table 5:**
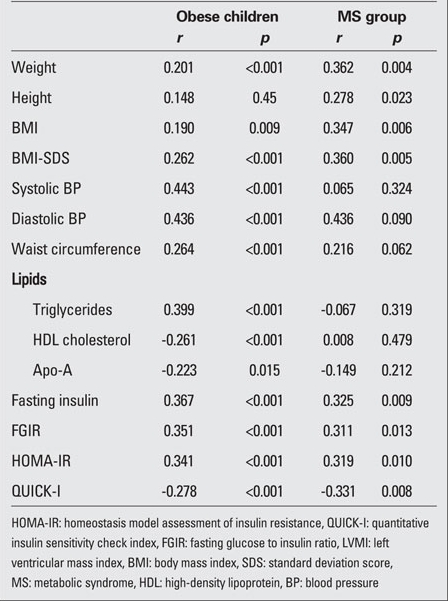
Table 5. Relationship between LVMI, insulin sensitivity indices, and other cardiovascular risk factors in obese children and in the MS group

**Table 6:**
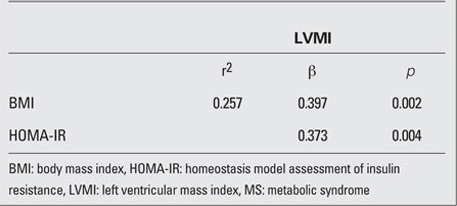
Table 6. Variables independently associated, in an age-, sex-, and other risk factor-adjusted backward multiple linear regression analysis, with the dependent variable LVMI in patients with MS

## DISCUSSION

In this study, we compared the LVMI between  obese patients and control subjects, and between patients within the obese group with MS and without MS, using echocardiographic measurements. In this study, we also  evaluated the effect of insulin resistance on LVMI in both obese children and children with MS.   

Obesity is associated with a group of metabolic  abnormalities (e.g., dyslipidemia, insulin resistance,  hyperglycemia, and hypertension) referred to as the MS. It has been suggested that the metabolic abnormalities could be involved in the modulation of LV structure. Several studies have investigated the relationship between insulin resistance and LV mass in both obese and hypertensive subjects with varying results (11,12,13). Some studies found a relation between insulin resistance and LVM (21,22), whereas others found no relation at all after adjusting for covariates. In the present study, the number of risk factors identified in both obese patients and those with MS was associated with increased LVMI. We observed that subjects with MS, diagnosed in accordance with the IDF consensus definition of MS in children and adolescents, exhibited higher LVMI values than subjects without MS.  Moreover, the relationship between MS and LVM was  confirmed in linear regression models including MS together with its individual components, as independent variables; this suggests that MS may have a harmful effect on myocardial structure over and above the potential contribution of each  single component of this syndrome, above all body size and BP, well-known independent determinants of LVM (22). 

Childhood obesity is a strong predictor of adult adiposity and is associated with increased levels of LVM, which is viewed as an important cardiovascular risk factor (23). Previous studies performed in adolescents and young adults have shown a strong association between BMI and high levels of LV mass, independent of systolic BP (24). 

 The association of MS with cardiac hypertrophy might  be explained also by nonhaemodynamic factors, such as  insulin resistance and the accompanying compensatory  hyperinsulinemia, which are considered to be the major  pathophysiological features underlying the MS (25). Trophic effects of insulin on myocardial tissue have been demonstrated in cell cultures and animal models and could be mediated,  at least in part, by the insulin-like growth factor-1 (IGF-1) receptors (26,27).  

A more central deposition of fat (i.e. android type) has been shown to be associated with increased triglycerides, decreased HDL cholesterol, increased systolic BP, and increased LVM in adolescents. In our study, the univariate analysis demonstrated that the LVMI was significantly associated with insulin  sensitivity indices derived from fasting samples.   Moreover, fasting insulin was significantly associated with LVMI in  children with obesity. Therefore, these data supported the idea that insulin resistance may have a role in the development of cardiac hypertrophy in obese children. 

In overweight adults, insulin resistance was linked to increased LVM independent of BMI and BP. The method of correcting LVM for body size and the criteria used to define LVM have varied between studies (21,23). LVM has been adjusted for height, body surface area, weight, and height raised to various powers. Because of the rise in the prevalence of obesity, indexing of LVM to weight or body surface area may allow an increased LVM to be interpreted erroneously as  normal (28). Height2.7 (in meters) has been validated as an  indicator of lean body mass and has been recommended for indexing LVM. Use of height2.7 to index LVM also minimizes the effect of age, gender, and race (23,24). A clamp study of adults showed no significant relationship between LVM and insulin resistance when obesity, BP, sex, and age were  covaried (28). However, the authors acknowledged that insulin resistance may mediate the influence of obesity on cardiac structure. Adjusting for obesity in studies of insulin resistance may not be appropriate, because of its collinearity with insulin. To our knowledge, this is the first study that gives the cut-off level of LVMI in children and adolescents with MS. The present LVMI cut-off point of 33g/m2 for the diagnosis of MS yielded a sensitivity of 97% and a specificity of 98%. 

A few pediatric studies have shown that LVM is related to insulin resistance syndrome characteristics in youth. Cross- sectional and longitudinal studies of normotensive young  people (6-27 years old) have revealed that LVM is predicted by adiposity and resting and challenge hemodynamics. Daniels et al (24) reported that in children 9 to 17 years old, LVM was more closely related to central fat than overall adiposity, and that  central fat was associated with less favorable lipid and BP  levels. Gutin et al (29) studied children 7 to 13 years old and found LVM to be related to percent body fat and insulin levels. The Bogalusa Heart Study reported an association between fasting blood sugar and LVM in youth (13–27 years old) (30). They showed that increased insulin levels predict greater LVM in obese persons, independent of relations among insulin,  obesity, and BP.   

Davis et al (30) examined clustering of insulin resistance syndrome characteristics and LVMI in a sample of healthy youths. LVMI was associated with higher fasting glucose, hyperinsulinemia, and central adiposity, as shown by increased waist girth. After accounting for contributions of race, gender, and systolic BP, both insulin and waist girth independently  predicted LVMI. A one-factor model of insulin resistance  syndrome including insulin, glucose, waist girth, and LVMI showed good fit to the data.  

These results show that an incipient cardiovascular risk  syndrome is detectable even in healthy young people. Cardiac structure seems to be as closely associated with this syndrome as fasting glucose level. These relationships among central  obesity, glycemia, hyperinsulinemia, and cardiac structure found in healthy youths suggest that alterations in cardiac  structure early in life may be part of the progression of insulin resistance syndrome pathophysiology, with important  implications for development of risk factors for hypertension, T2DM, and atherosclerosis. In the present study, evaluation of obese children showed that fasting glucose was not  associated with LVMI, although fasting insulin was significantly associated with LVMI. Therefore, these data supported the idea that insulin resistance may have a role in the increasing LVMI in children with obesity. In the present study, conducted in a group of obese children, we found that insulin sensitivity indices derived from fasting samples and elevated basal insulin levels were significantly associated with increased LVMI.  

 Many of the previous studies evaluating LVM in children were performed in years prior to the dramatic increase which occurred in prevalence of obesity (31). Hanevold et al (32) found that the prevalence of LV hypertrophy in a multiethnic group of children and adolescents with hypertension was 15.5% using adult criteria and 41.1% using pediatric criteria, and that increasing BMI was associated with a higher LVMI. However, this study suggested that LV hypertrophy occurs commonly in children with hypertension and is associated with an increased BMI.  

In conclusion, we think that our optimal LVMI cut-off value for identifying MS may be a sensitive index in screening obese children and adolescents with pediatric MS. We suggest that assessment of LVMI in routine echocardiographic examinations of obese children and  dolescents might be used in predicting the presence of MS and its associated cardiovascular risks.  MS seems to increase LVM over and above the potential  contribution of BP, body size and other single components of this syndrome. Since LVM is a well-known predictor of  cardiovascular events, our results may partly explain the enhanced cardiovascular risk associated with MS.

## References

[1] Atabek ME, Pirgon O, Kurtoglu S (2006). Prevalence of metabolic syndrome in obese Turkish children and adolescents. Diabetes Res Clin Pract.

[2] Berenson GS (2002). Childhood risk factors predict adult risk associated with subclinical cardiovascular disease. The Bogalusa Heart Study Am J Cardiol.

[3] Srinivasan SR, Bao W, Wattigney WA, Berenson GS (1996). Adolescent overweight is associated with adult overweight and related multiple cardiovascular risk factors. The Bogalusa Heart Study. Metabolism.

[4] Boa W, Srinivasan SR, Wattigney WA, Berenson GS (1994). Persistence of multiple cardiovascular risk clustering related to syndrome X from childhood to young adulthood. Arch Intern Med.

[5] Giordano U, Ciampalini P, Turchetta A, Santilli A, Calzolari F, Crinò A, Pompei E, Alpert BS, Calzolari A (2003). Cardiovascular hemodynamics: relationships with insulin resistance in obese children. Pediatr Cardiol.

[6] Reaven GM (989). Role of insulin resistance in human disease. Diabetes.

[7] DeFronzo RA, Ferrannini E (1991). Insulin resistance: a multifaceted syndrome responsible for NIDDM, obesity, hypertension, dyslipidemia, and atherosclerotic cardiovascular disease.. Diabetes Care.

[8] Stern MP (1995). Diabetes and cardiovascular disease. The "common soil" hypothesis.. Diabetes.

[9] Decsi T, Molnar D (2003). Insulin resistance syndrome in children. Pediatr Drugs.

[10] Galli-Tsinopoulou A, Karamouzis M, Arvanitakis SN (2003). Insulin resistance and hyperinsulinemia in prepubertal obese children. J Pediatr Endocrinol Metab.

[11] Ilercil A, Devereux RB, Roman MJ, Paranicas M, O'Grady MJ, Lee ET, Welty TK, Fabsitz RR, Howard BV (2002). Associations of insulin levels with left ventricular structure and function in American Indians: the strong heart study. Diabetes.

[12] Atabek ME, Pirgon O, Kivrak AS (2007). Evidence for association between insulin resistance and premature carotid atherosclerosis in childhood obesity. Pediatr Res.

[13] Atabek ME, Pirgon O, Kurtoglu S (2007). Assessment of abnormal glucose homeostasis and insulin resistance in Turkish obese children and adolescents. Diabetes Obes Metab.

[14] 14.	Kuczmarski RJ, Ogden CL, Guo SS, Grummer-Strawn LM, Flegal KM, Mei Z, Wei R, Curtin LR, Roche AF, Johnson CL
 (2002). 2000 CDC Growth Charts for the United States: methods and development. Vital Health Stat 11.

[15] Hatipoglu N, Ozturk A, Mazicioglu MM, Kurtoglu S, Seyhan S, Lokoglu F (2008). Waist circumference percentiles for 7-to 17-year-old Turkish children and adolescents. Eur J Pediatr.

[16] Matthews DR, Hosker JP, Rudenski AS, Naylor BA, Treacher DF, Turner RC (1985). Homeostasis model assessment: insulin resistance and beta-cell function from fasting plasma glucose and insulin concentrations in men. Diabetologia.

[17] Katz A, Nambi SS, Mather K, Baron AD, Follmann DA, Sullivan G, Quon MJ (2000). Quantitative insulin sensitivity check index: a simple, accurate method for assessing insulin sensitivity in humans. J Clin Endocrinol Metab.

[18] Feigenbaum H (1994). Echocardiography, Fifth ed.

[19] Devereux RB, Alonso DR, Lutas EM, Gottlieb GJ, Campo E, Sachs I, Reichek N (1986). Echocardiographic assessment of left ventricular hypertrophy: comparison to necropsy findings. Am J Cardiol.

[20] Zimmet P, Alberti KGMM, Kaufman F, Tajima N, Silink M, Arslanian S, Wong G, Bennett P, Shaw J, Caprio S; IDF Consensus Group (2007). The metabolic syndrome in children and adolescents – an IDF consensus report. Pediatric Diabetes.

[21] Chinali M, de Simone G, Roman MJ, Lee ET, Best LG, Howard BV, Devereux RB (2006). Impact of obesity on cardiac geometry and function in a population of adolescents: the Strong Heart study. J Am Coll Cardiol.

[22] Chinali M, de Simone G, Roman MJ, Best LG, Lee ET, Russell M, Howard BW, Devereux RB (2008). Cardiac markers of pre-clinical disease in adolescents with the metabolic syndrome: the strong heart study. J Am Coll Cardiol.

[23] de Simone G, Daniels SR, Devereux RB, Meyer RA, Roman MJ, de Divitiis O, Alderman MH (1992). Left ventricular mass and body size in normotensive children and adults: assessment of allometric relations and impact of overweight. J Am Coll Cardiol.

[24] Daniels SR, Witt SA, Glascock B, Khoury PR, Kimball TR (2002). Left atrial size in children with hypertension: the influence of obesity, blood pressure, and left ventricular mass. J Pediatr.

[25] Azevedo A, Bettencourt P, Almeida PB, Santos AC, Abreu-Lima C, Hense HW, Barros H (2007). Increasing number of components of the metabolic syndrome and cardiac structural and  functional abnormalities--cross-sectional study of the general populatio. BMC Cardiovasc Disord.

[26] Strauss DS (1984). Growth-stimulatory actions of insulin in vitro and in vivo. Endocr Rev.

[27] Holmäng A, Yoshida N, Jennische E, Waldenström A, Björntorp P (1996). The effects of hyperinsulinaemia on myocardial mass, blood pressure regulation and central haemodynamics in rats. Eur J Clin Invest.

[28] Malmqvist K, Ohman KP, Lind L, Nyström F, Kahan T (2002). Relationships between left ventricular mass and the renin–angiotensin system, catecholamines, insulin and leptin. J Intern Med.

[29] Davis CL, Kapuku G, Snieder H, Kumar M, Treiber FA (2002). Insulin resistance syndrome and left ventricular mass in healthy young people. Am J Med Sci.

[30] Gutin B, Treiber F, Owens S, Mensah GA (1998). Relations of body composition to left ventricular geometry and function in children. J Pediatr.

[31] Urbina EM, Gidding SS, Bao W, Elkasabany A, Berenson GS (1999). Association of fasting blood sugar level, insulin level, and obesity with left ventricular mass in healthy children and adolescents: The Bogalusa Heart Study. Am Heart J.

[32] Hanevold C, Waller J, Daniels S, Portman R, Sorof J (2004). International Pediatric Hypertension Association. The effects of obesity, gender, and ethnic group on left ventricular hypertrophy and geometry in hypertensive children: a collaborative study of the International Pediatric Hypertension Association. Pediatrics.

